# Quantitative Evaluation of Post-stenotic Blood Flow Disturbance in Canine Femoral Artery Stenosis Model: An Early Experience With Vector Flow Imaging

**DOI:** 10.3389/fcvm.2022.829825

**Published:** 2022-02-24

**Authors:** Rui Zhao, Haining Zheng, Wei Wang, Yigang Du, Yisha Tong, Chaoyang Wen

**Affiliations:** ^1^Department of Ultrasound, Peking University International Hospital, Beijing, China; ^2^Department of Ultrasound, The Fourth Medical Center of PLA General Hospital, Beijing, China; ^3^Shenzhen Mindray Bio-Medical Electronics Co., Ltd., Shenzhen, China; ^4^Department of Vascular Surgery, Austin Hospital, University of Melbourne, Melbourne, VIC, Australia

**Keywords:** arterial stenosis, Doppler, duplex ultrasound, femoral artery, post-stenotic turbulence, vector flow imaging

## Abstract

**Objective:**

To investigate the value of Vector Flow Imaging (V Flow) in the assessment of post-stenotic turbulence in the canine arterial stenosis model.

**Materials and Methods:**

Canine femoral artery stenosis models were established using ameroid constrictors in 12 beagle dogs. 50% and then 70% femoral artery stenoses were confirmed by selective femoral artery angiography. V Flow was used to measure femoral artery flow turbulence index (Tur) preoperatively as a baseline. After establishing of a 50% and then 70% stenoses, the Tur indices were recorded in the femoral artery at 1, 3, 5, 7, 9, 11, 13, 15, 17, and 19 mm distal to the stenosis.

**Results:**

Baseline Tur indices of normal canine femoral arteries were <1% in 11 of 12 cases (91.7%). Distal to a 50% stenosis, the Tur index (>1%) was recorded in 83.3–100% cases between 1 and 9 mm, 41.7–58.3% between 11 and 17 mm, and 16.7% at 19 mm. For a 70% stenosis, the Tur index (>1%) occurred in 81.8–100% cases between 1 and 17 mm distal to the stenosis, and 63.6% at 19 mm. The Tur index peaked around 7 mm or 2.3 times of the initial vessel diameter (3 mm) downstream for a 50% stenosis and 11 mm or 3.7 times of vessel diameter downstream for a 70% stenosis.

**Conclusion:**

V Flow with Tur index measurement adds quantitative information of post-stenotic turbulence when assessing an arterial stenosis with ultrasound. Tur index of 1% seems a useful threshold for assessment of flow turbulence in this small sample study. Further studies with larger sample size are needed to evaluate the value of V Flow in clinical applications.

## Introduction

Arterial stenosis/occlusion is a common vascular pathology and most commonly caused by atherosclerosis (1, 2). Other causes of this condition include Takayasu's disease, Burger's disease (thromboangiitis obliterans), fibromuscular dysplasia, adventitial cystic disease of the popliteal artery, popliteal artery entrapment, endofibrosis of the external iliac artery (iliac artery syndrome in cyclists), thoracic outlet syndrome, etc. (2, 3). Depending on the artery or arteries involved, arterial stenosis/occlusion may result in ischaemia of the brain, viscera, upper or lower limbs and cause relevant ischaemic symptoms.

Duplex ultrasound scanning has been widely used to evaluate arterial stenosis in various parts of the human body, including carotid, upper limb, visceral/renal, aortoiliac and lower limb arteries (4–6). Duplex ultrasound assessment of arterial stenosis includes observation of intraluminal plaque, measurement of velocities and evaluation of post-stenotic turbulence, as well as the profile of the waveform (7). Traditionally, flow turbulence is qualitatively assessed by the appearance of multiple colors on the color Doppler image and spectral broadening on the spectral Doppler waveform. On the spectral Doppler waveform, the spectral window is the black zone between the spectral line and the baseline. Spectral broadening is widening of the spectral line and filling of the spectral window (8). However, neither the color Doppler image nor the spectral Doppler waveform assessment is quantitative. Recently, high frame rate vector flow imaging (V Flow) has become available (9–11) and it is capable to quantitatively evaluate flow turbulence (11, 12). Most published studies using V Flow have been investigations on carotid arteries for blood flow visualization, wall shear stress measurement and volume flow estimation, as well as in the assessment of arterial stenosis before and after carotid artery stenting (13–21).

## Materials and Methods

The experiment study was performed between April 2020 and July 2020. The study was approved by the PLA General Hospital Medical Ethics Committee.

### Establishment of Canine Femoral Artery Stenosis Model

Twelve beagle dogs aged 12–24 months were purchased from the Animal Experiment Centre of Nongnong (Beijing) Biotechnology Co. Ltd. (Beijing, China). Preoperative duplex ultrasound scanning was performed to confirm that internal diameters of the canine femoral arteries were 3.0 ± 0.1 mm.

The aim of this study is to explore the value of V Flow in the evaluation of post-stenotic turbulence in the canine arterial stenosis model.

Ameroid constrictors with inner diameters of 2.5 mm (Research Instruments SW, USA) were used to create canine femoral artery stenosis. An ameroid constrictor has an inner ring of casein that is surrounded by a stainless-steel sheath. Casein is a hygroscopic material that swells as it slowly absorbs body fluid and the stainless-steel sheath forces the casein to swell inwardly. This results in the internal diameter of the constrictor decreasing gradually over a few weeks.

Under general anesthesia, the right canine femoral artery in the groin was dissected and applied with 3 ml lidocaine-papaverine solution (50 mg/2.5 ml lidocaine and 15 mg/0.5 ml papaverine) to prevent vasospasm. An ameroid constrictor ring was then placed over the artery.

**Figure 1:**
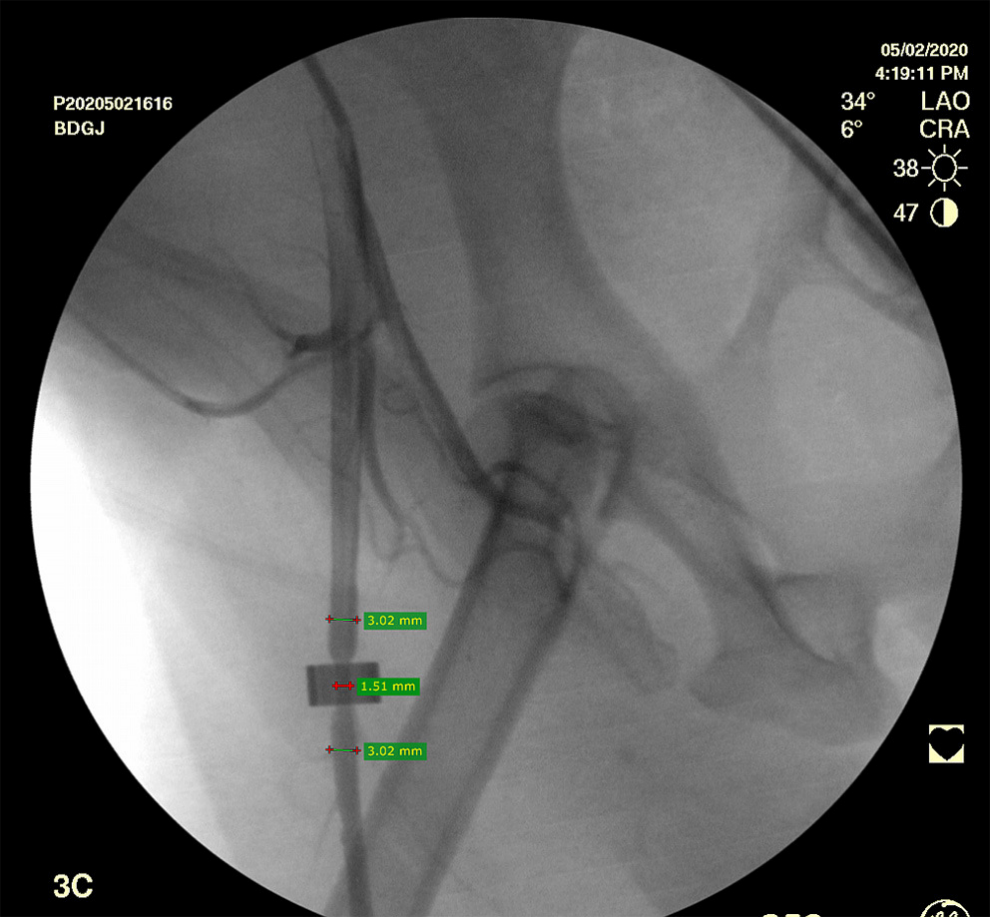
Selective right femoral artery angiogram shows a canine femoral artery stenosis (50%).

An arterial duplex ultrasound scan was performed 4–5 h after the surgery to exclude femoral artery spasm. A selective right femoral artery angiogram with was then performed with 2 perpendicular projections via left femoral artery catherization (OEC 9900 Elite mobile C-arm imaging system, GE Healthcare, USA). The establishment of the 50% canine femoral artery stenosis using the constrictor ring was confirmed when the angiogram demonstrated 50% stenosis (45–52%) (Figure 1).

Post-operative arterial duplex ultrasound scan was repeated at 7–8 days. Peak systolic velocity ratio (PSV at the stenosis / PSV proximal to the stenosis) of 4 indicates a 70% stenosis. Confirmation of 70% canine femoral artery stenosis was obtained when the right femoral angiogram demonstrated an arterial stenosis of 68–72% at the site of the constrictor ring.

### V Flow Ultrasound Assessment of Canine Femoral Artery

**Figure 2:**
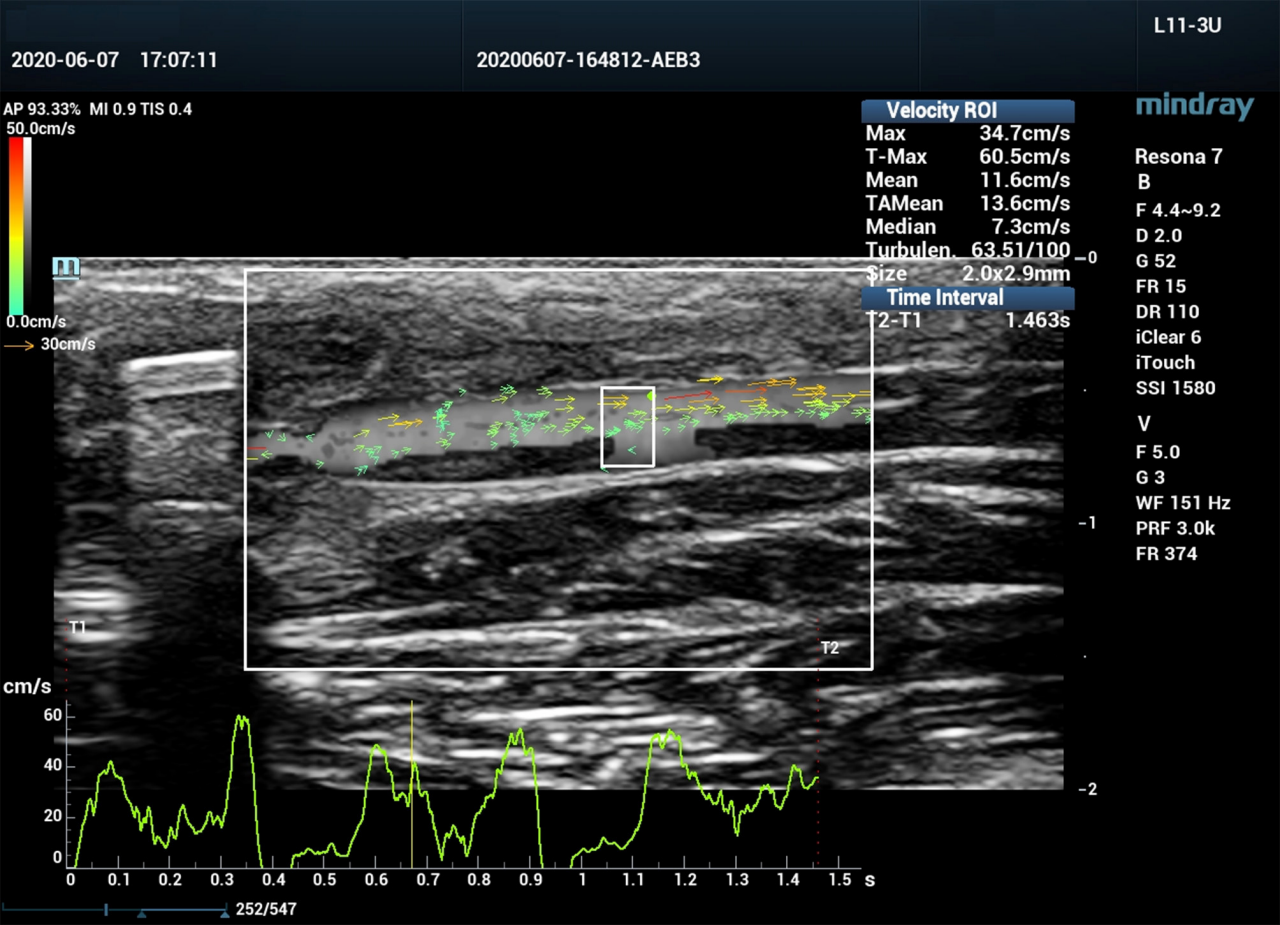
Vector flow imaging (V Flow) of a canine femoral artery.

All ultrasound assessments were performed with a Mindray Resona 7 ultrasound system (Shenzhen Mindray Bio-Medical Electronics Co., Ltd., Shenzhen, China). A 3-11 MHz linear array transducer (L11-3U) was used for the examination. The ultrasound system was equipped with V Flow mode, and is capable of obtaining blood flow turbulence index (Tur) at regions of interest (ROI). V Flow uses a series of arrows to represent blood flow direction and velocity. The arrow orientation indicates the direction of blood flow, and the arrow length and color represent the flow velocity. The longer the arrow, the higher the velocity. The range of velocities from high to low are expressed by colors from red to orange, to yellow, and to green (Figure 2). Tur indices were calculated by the ultrasound system using the following equation:


$$Tur=\left(1-\frac{\sqrt{C^{2}+S^{2}}}{N}\right)\times 100\%$$

Where


$$\begin{array}{l} C=\overset{N}{\underset{i=1}{\sum }}\cos\theta_{i}\\ S=\overset{N}{\underset{i=1}{\sum }}\sin\theta_{i} \end{array}$$

*N*: number of velocity measuring points in the ROI, θ_*i*_: flow velocity angle at *i*th measuring point, and Tur indices ranged from 0 to 1 (0–100%), 0 indicates pure laminar flow which means that all flow directions are exactly the same. The bigger the Tur is, the more turbulent the flow is (11).

#### V Flow Acquisition

V Flow ultrasound images of the right femoral artery in all 12 dogs were recorded preoperatively as a baseline and then after the placement of an ameroid constrictor when the femoral artery stenosis reached 50% and then 70%. Grayscale ultrasound was used first to display the longitudinal view of the femoral artery. The “V Flow” tab was activated to enter the V Flow mode and 1.5 s of V Flow cine loop was captured with image width of 2.4 cm, depth of 1.5 cm and arrow density of 10%. The heart rate (HR) of the experimental dog was simultaneously recorded.

#### Tur Index Measurement

A V Flow cine loop recorded in the ultrasound system was uploaded, and then the “Velocity ROI” box was positioned to the predefined area of the femoral artery for Tur index measurement. The width of the ROI was 2 mm and the depth was the internal diameter of the artery. Tur indices were measured preoperatively, and then 1, 3, 5, 7, 9, 11, 13, 15, 17, and 19 mm distal to the femoral artery stenosis at 50% and then 70%. Export Tur index data of 1.5-s V Flow cine loop (~2–3 cardiac cycles) to a .CVS file containing 500-600 Tur indices, each was from one of 500–600 V Flow frames.

#### Tur Index Calculation

Tur indices at different cardiac phases are different even in the same ROI (10) and it is not always possible to determine each frame of the V Flow image captured from the specific moment of a cardiac cycle. Therefore, the averaged Tur index during one cardiac cycle from the beginning of the recorded V Flow cine loop was calculated for each observation. Number of frames in one cardiac cycle (N) is calculated based on the V Flow frame rate (FR) and the HR at the time:


$$\begin{array}{l} N=\text{FR}/\left(\text{HR}/60\right)=60\times \text{ FR}/\text{HR}. \end{array}$$

The mean Tur index from one cardiac cycle was calculated by the sum of Tur indices from the first frame to *N*th frame divided by *N*.

## Results

Diameters of the right canine femoral arteries in 12 beagle dogs were 2.9–3.1 mm based on preoperative duplex ultrasound assessment.

Baseline Tur indices obtained from the femoral artery prior to the placement of the ameroid constrictor were shown in Table 1. The Tur index <1% was in 11 of 12 arteries (91.7%).

**Table 1:** Baseline Tur indices (%) in 12 canine femoral arteries.

**Case No**	**Tur indices (%)**	**Mean**	**Standard deviation**	**No of cases with Tur <1% (%)**
1	0.18	0.38	0.68	11 (91.7)
2	0.12			
3	0.24			
4	0.25			
5	0.99			
6	0.04			
7	0.04			
8	0.11			
9	0.09			
10	2.38			
11	0.07			
12	0.02			

**Figure 3:**
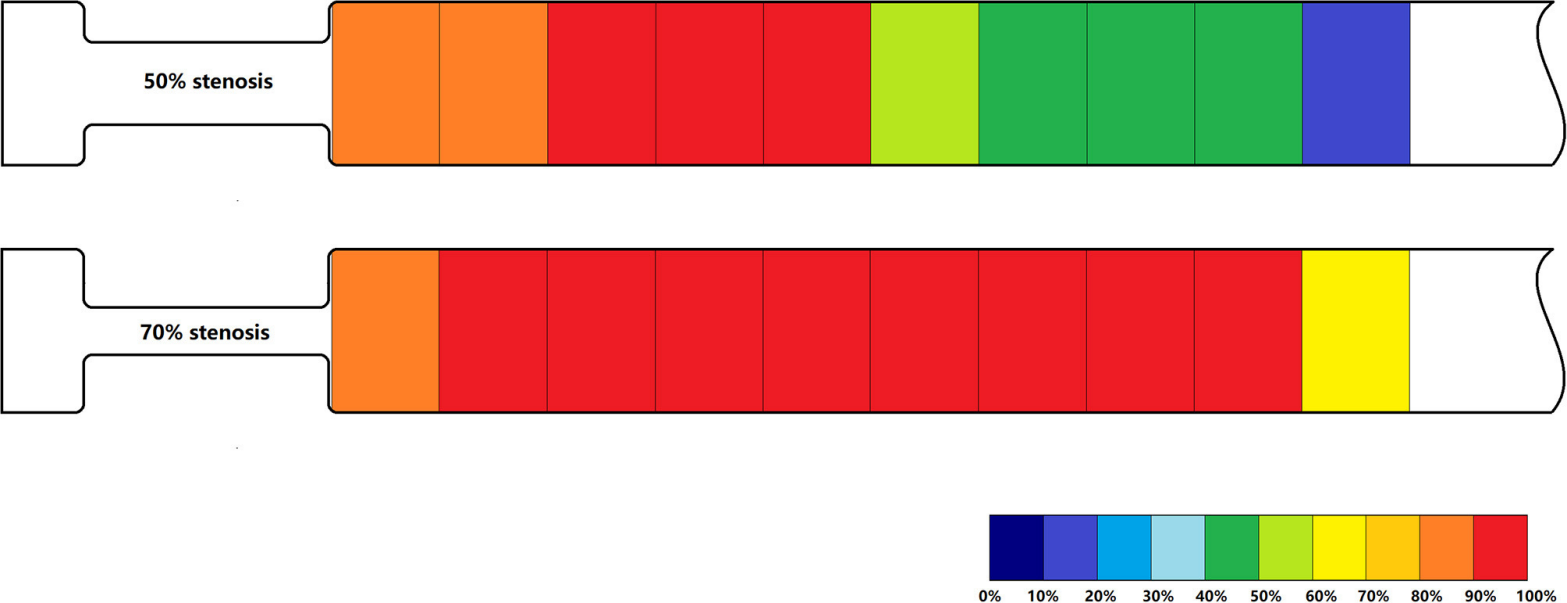
Percentage of Tur indices (>1%) in relation to distances distal to a 50% stenosis (above) and a 70% stenosis (below) in 12 canine femoral arteries.

**Figure 4:**
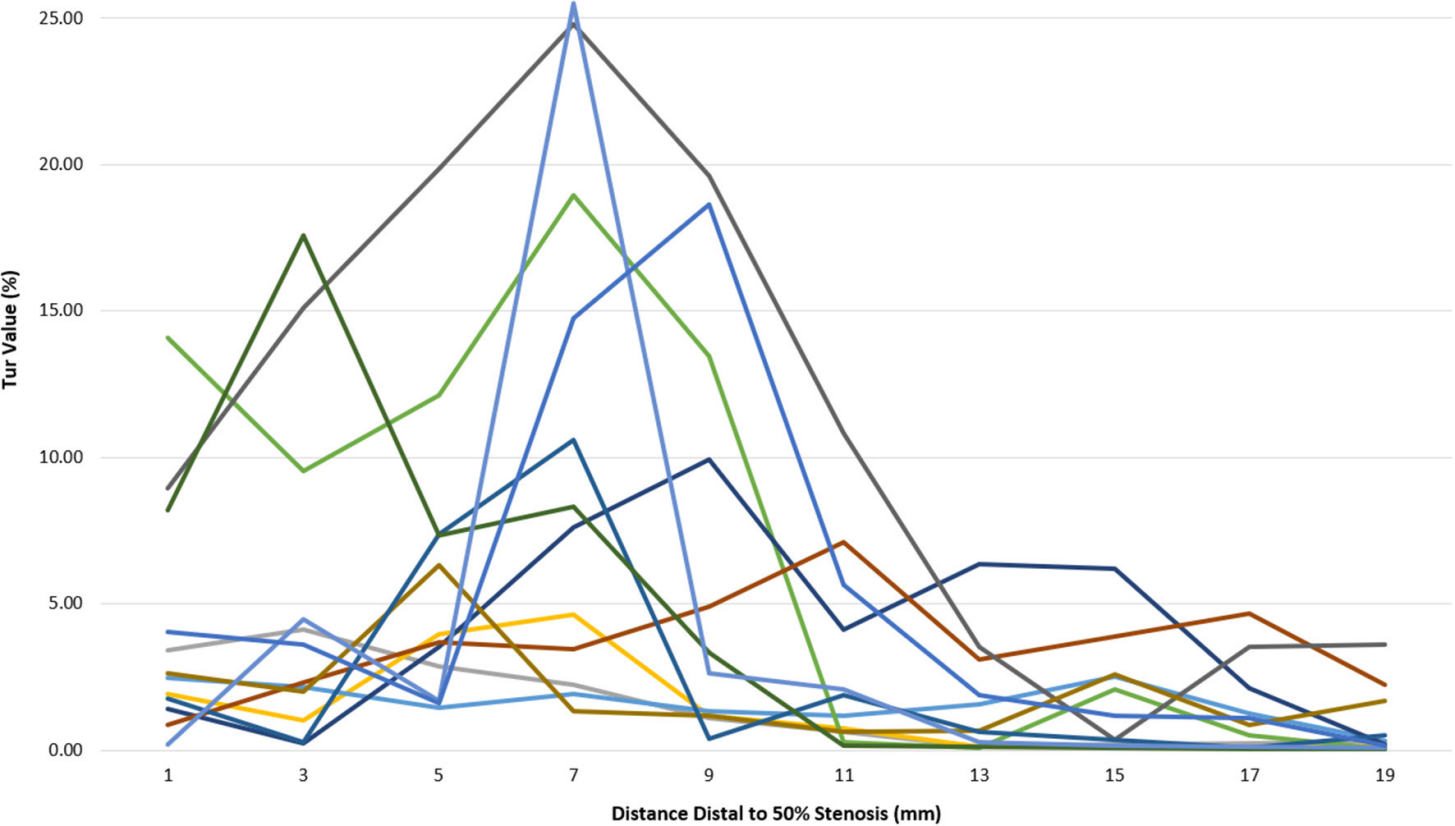
Tur index changes in relation to distance distal to a 50% stenosis in 12 canine femoral arteries.

Post-stenotic Tur indices recorded between 1 and 19 mm distal to a 50% stenosis following ameroid constrictor placement in 12 canine femoral arteries are shown in Table 2. The Tur index (>1) occurred in 83.3–100% of the cases between 1 and 9 mm distal to the stenosis. The number of cases with Tur index (>1%) decreased to 41.7–58.3% between 11 and 17 mm distal to the stenosis, and only 16.7% at 19 mm (Table 2 and Figure 3). Figure 4 shows Tur index changes in relation to distances distal to a 50% stenosis. Based on a canine femoral artery diameter of 3 mm, post-stenotic Tur index increased immediately distal to the stenosis and reached its maximum value at 5–11 mm or 1.7–3.7 diameters downstream in 9 of 12 cases (75%) with most at 7 mm or 2.3 times the vessel diameter downstream (5/12). The Tur index decreased sharply afterwards (Table 2 and Figure 4).

**Table 2:** Tur indices (%) distal to a 50% stenosis in 12 canine femoral arteries.

**Distance distal to stenosis (mm)**		**1**	**3**	**5**	**7**	**9**	**11**	**13**	**15**	**17**	**19**
Tur (%)	No 1	3.42	4.12	2.85	2.25	1.11	0.62	0.14	0.19	0.25	0.30
	No 2	1.94	1.03	3.97	4.63	1.19	0.74	0.13	0.11	0.09	0.15
	No 3	2.49	2.16	1.45	1.94	1.35	1.17	1.56	2.52	1.24	0.33
	No 4	14.09	9.52	12.11	18.96	13.45	0.28	0.06	2.07	0.50	0.05
	No 5	1.43	0.23	3.54	7.62	9.91	4.11	6.36	6.20	2.10	0.20
	No 6	0.87	2.31	3.69	3.46	4.89	7.11	3.12	3.89	4.65	2.22
	No 7	8.95	15.11	19.85	24.81	19.61	10.83	3.52	0.36	3.52	3.59
	No 8	2.62	2.00	6.33	1.34	1.18	0.64	0.67	2.60	0.86	1.70
	No 9	1.77	0.28	7.36	10.60	0.40	1.88	0.62	0.35	0.09	0.52
	No 10	8.20	17.56	7.33	8.33	3.32	0.14	0.12	0.08	0.06	0.06
	No 11	0.21	4.47	1.70	25.49	2.64	2.07	0.26	0.17	0.12	0.08
	No 12	4.03	3.61	1.63	14.75	18.63	5.64	1.88	1.19	1.09	0.14
Mean		4.17	5.20	5.98	10.35	6.47	2.94	1.54	1.64	1.21	0.78
Standard deviation		4.14	5.78	5.37	8.74	7.11	3.36	1.93	1.91	1.49	1.13
No of cases with Tur <1% (%)		2 (16.7)	2 (16.7)	0 (0)	0 (0)	1 (8.3)	5 (42.7)	7 (58.3)	6 (50.0)	7 (58.3)	10 (83.3)
No of cases with Tur >1% (%)		10 (83.3)	10 (83.3)	12 (100)	12 (100)	11 (91.7)	7 (58.3)	5 (41.7)	6 (50.0)	5 (41.7)	2 (16.7)
No of cases with maximum Tur (%)		0 (0)	2 (16.7)	1 (8.3)	5 (41.7)	2 (16.7)	1 (8.3)	0 (0)	1 (8.3)	0 (0)	0 (0)

**Figure 5:**
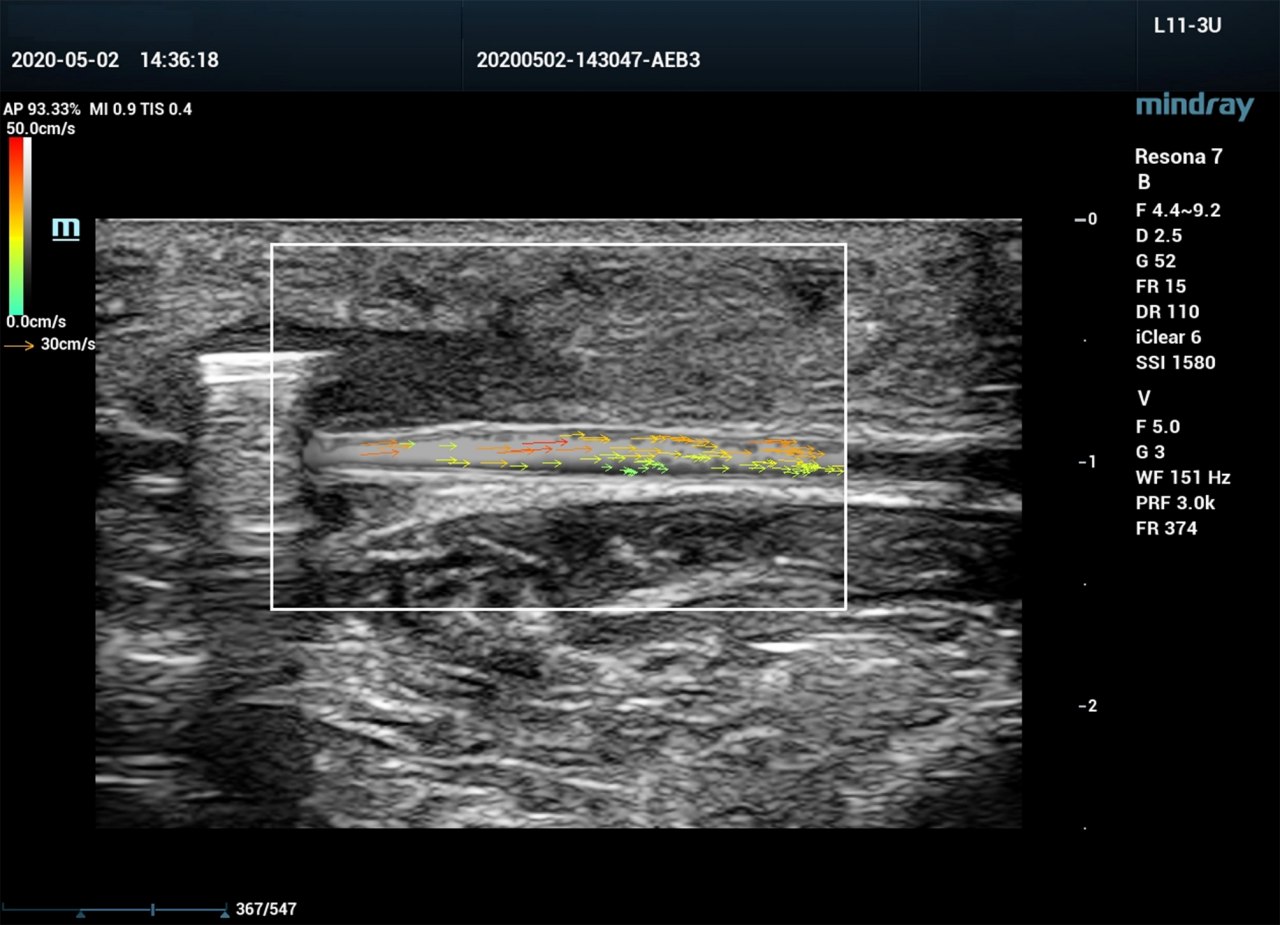
V Flow ultrasound image of a canine femoral artery with post-stenotic segment spasm.

**Figure 6:**
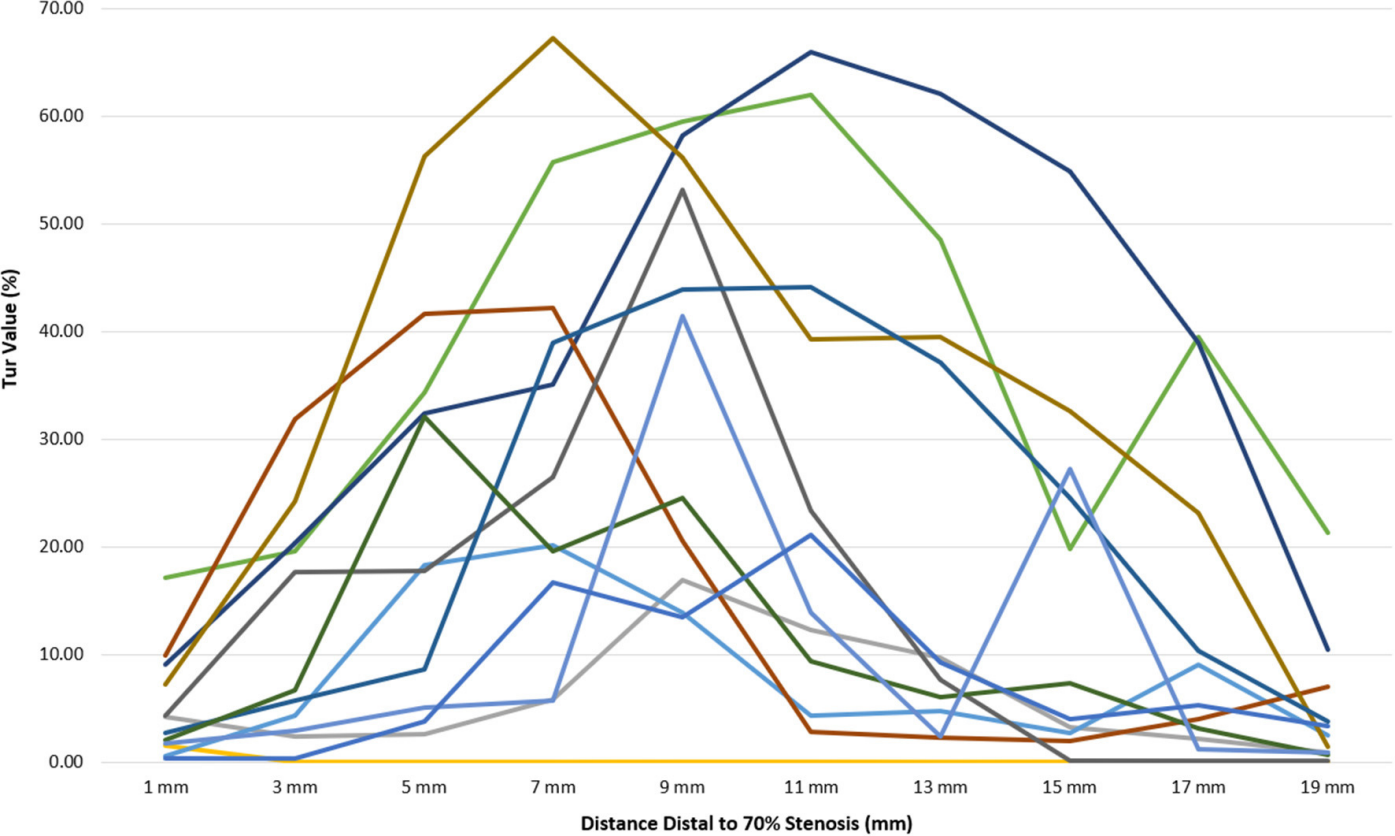
Tur index changes in relation to distance distal to a 70% stenosis in 12 canine femoral arteries.

Post-stenotic Tur indices distal to a 70% stenosis are shown in Table 3. Post-stenotic segment spasm occurred in Case 2. The PSVs in the segment were ranged from 128 cm/s to 198 cm/s, and the Tur index (<1%) occurred in 9 of 10 measurement sites (Table 3 and Figure 5). This case is not included in the calculation for mean, standard deviation, number of cases with Tur <1%, number of cases with Tur>1%, and number of cases with maximum Tur. In the remainder of 11 cases, the Tur index (>1%) was 81.8–100% between 1 and 17 mm distal to the stenosis, and 63.6% at 19 mm (Table 3 and Figure 3). Figure 6 shows Tur index changes in relation to distances distal to a 70% stenosis. Based on a canine femoral artery diameter of 3 mm, post-stenotic Tur index increased immediately distal to the stenosis and reached its maximum value at 5–11 mm or 1.7–3.7 diameters downstream in all 11 cases (100%) followed by a decrease (Table 3 and Figure 6).

**Table 3:** Tur indices (%) distal to a 70% stenosis in 12 canine femoral arteries.

**Distance distal to** **stenosis (mm)**		**1**	**3**	**5**	**7**	**9**	**11**	**13**	**15**	**17**	**19**
Tur (%)	No 1	4.19	2.34	2.58	5.83	16.95	12.30	9.66	3.21	2.15	0.91
	No 2^*^	1.49	0.05	0.03	0.03	0.01	0.01	0.02	0.01	0.02	0.03
	No 3	0.55	4.25	18.26	20.16	13.84	4.26	4.73	2.73	9.07	2.46
	No 4	17.08	19.59	34.29	55.76	59.48	61.97	48.49	19.83	39.47	21.31
	No 5	9.08	20.33	32.39	35.09	58.24	66.02	62.10	54.93	38.95	10.44
	No 6	9.94	31.85	41.67	42.24	20.57	2.84	2.31	2	4.01	6.96
	No 7	4.26	17.6	17.76	26.51	53.19	23.31	7.66	0.08	0.12	0.09
	No 8	7.25	24.21	56.23	67.26	56.19	39.24	39.53	32.59	23.11	1.4
	No 9	2.73	5.68	8.60	38.97	43.89	44.08	37.16	24.57	10.38	3.78
	No 10	2.05	6.64	32.13	19.62	24.54	9.41	5.99	7.35	3.09	0.61
	No 11	1.76	2.86	5.09	5.67	41.42	13.89	2.38	27.19	1.13	0.87
	No 12	0.31	0.33	3.74	16.68	13.43	21.15	9.26	4.02	5.32	3.32
Mean^**^		5.38	12.33	22.98	30.34	36.52	27.13	20.84	16.23	12.44	4.74
Standard deviation^**^		5.08	10.69	17.66	19.69	18.90	22.43	21.63	17.35	14.69	6.32
No of cases with Tur <1% (%)^**^		2 (18.2)	1 (9.1)	0 (0)	0 (0)	0 (0)	0 (0)	0 (0)	1 (9.1)	1 (9.1)	4 (36.4)
No of cases with Tur >1% (%)^**^		9 (81.8)	10 (90.9)	11 (100)	11 (100)	11 (100)	11 (100)	11 (100)	10 (90.9)	10 (90.9)	7 (63.6)
No of cases with maximum Tur^**^		0 (0)	0 (0)	1 (9.1)	3 (27.3)	3 (27.3)	4 (36.4)	0 (0)	0 (0)	0 (0)	0 (0)

* *Post-stenotic segment spasm occurred in Case 2*.

** *Case 2 is excluded from calculation*.

**Figure 7:**
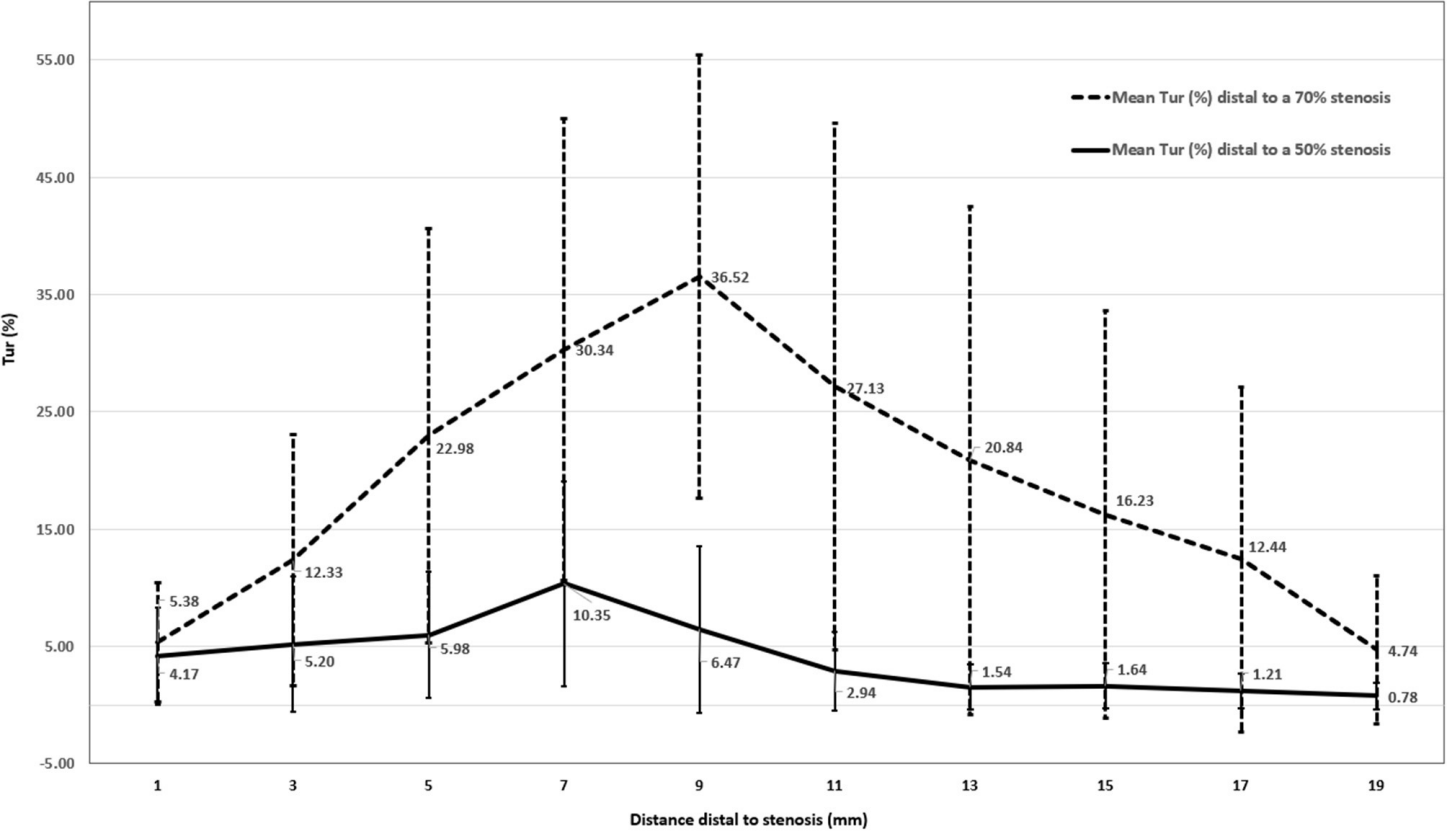
Mean Tur index changes distal to a 50% stenosis (*N* = 12) and a 70% stenosis (*N* = 11) in canine femoral arteries (error bars represents standard deviation of the means).

Figure 7 displays mean Tur index changes distal to a 50% stenosis (solid line) and a 70% stenosis (dot line). It appears that post-stenotic Tur index increases were higher and lasted longer in arteries with a 70% stenosis than those with a 50% stenosis. It is needed to point out that Tur index values in both groups had a large standard deviation (see error bars in the figure).

## Discussion

Blood flow in a straight artery with near uniform diameters is generally considered to be close to laminar flow (22). Non-laminar flow in an artery can be observed when it becomes bifurcated, dilated, tortuous, downstream to a stenosis, or has an arteriovenous communication (23). It is believed that post-stenotic turbulence is related to post-stenotic dilation and wall shear stress (24). Since blood behaves as a non-Newtonian fluid and changes its viscosity when flow velocity changes, this further complicates flow turbulence, which has been investigated by a series of numerical studies (25–28). Evaluation of post-stenotic flow turbulence is important in the diagnosis of arterial stenosis, and included in many duplex ultrasound diagnostic criteria (8, 29, 30). The study of flow turbulence is also helpful in understanding of the mechanism of formation and progression of vascular disorders as well as in the design of medical devices that mimic or alter blood flow (31).

Conventional ultrasound systems are able to measure blood flow velocities with spectral Doppler and observe flow direction with color Doppler imaging. Spectral Doppler and color Doppler are based on Doppler principle and angle-dependency. Therefore, Doppler ultrasound only estimates the axial component of flow velocity and is limited by the vessel geometry. When flow turbulence is present in an artery, blood flows in multiple directions. These ultrasound systems can only provide qualitative information regarding flow turbulence by multiple colors on color Doppler imaging and spectral broadening on pulsed wave (PW) Doppler spectrum representing various flow velocities and multiple flow directions. However, evaluations of flow turbulence by both color Doppler imaging and PW Doppler spectrum are qualitative and have their limitations. If the color velocity scale is set below the mean velocity of blood flow, aliasing may appear throughout the vessel lumen that makes it difficult to identify flow turbulence on color Doppler. On the PW Doppler spectrum, spurious spectral broadening can result from a large Doppler angle, a large sample volume (>3.5 mm), a sample volume located close to the vessel wall, or a high PW Doppler gain setting (32). V Flow uses multi-directional ultrasound transmission and reception (9) based on plane wave and focus wave interleaved scanning (11). The actual direction of the velocity is reconstructed by the velocity components from different directions. Therefore, it is capable of visualizing and measuring flow velocities in all directions (14). The Tur index measurement makes it possible to assess flow turbulence quantitatively (11, 12). However, it is not fully understood how to apply the Tur index in the assessment of flow turbulence and its clinical significance.

Theoretically, pure laminar flow travels in parallel layers with exactly the same flow directions and the Tur index equals to 0% while most extra turbulent flow with opposite direction flow has a Tur index of 100% (11, 12). In this study, Tur index was <1% (0.02–0.99%) in 11 of 12 normal canine femoral arteries (91.7%). Tur index (>1%) was observed immediately distal to an arterial stenosis, in 10 of 12 cases (83%) distal to a 50% stenosis and 9 of 11 cases (82%) distal to a 70% stenosis. The Tur index peaked within 1.7–3.7 diameters downstream in 9 of 12 cases (75%) to a 50% stenosis and in all 11 cases (100%) to a 70% stenosis. At 19 mm or 6.3 diameters downstream to a stenosis, the Tur index recovered to <1% in more cases with a less significant stenosis (50%) than those with a more significant stenosis (70%), being 83.3% (10/12) vs. 36.4% (4/11).

There is no published threshold of the Tur index for distinguishing turbulent flow from normal flow in a straight artery. This study attempts to explore such a threshold. Tur index of 1% seems a useful threshold for assessment of flow turbulence in this small sample study. Further studies are needed to determine clinically significant thresholds for various clinical applications. Post-stenotic Tur index values had a large standard deviation in arteries with a 50% stenosis and with a 70% stenosis. Therefore, it is less meaningful to compare the mean Tur of the two groups. Nevertheless, it seems arteries with a more severe stenosis had higher and longer lasting post-stenotic Tur index values than those with a less severe stenosis.

It should be pointed out that the size of the canine femoral arteries studied was approximately 3 mm in diameter and much smaller than those most commonly studied human arteries such as carotid and femoropopliteal arteries. Haemodynamic changes including its response to a stenosis in those arteries might be different.

### Limitations

There are limitations in this study. Small sample size is an obvious one. Tur indices are not the same for the same ROI at different cardiac phases (11). When a stenosis is present, it is not always possible to determine the precise cardiac phase of a Tur index recorded based on the V Flow velocity waveform. Therefore, Tur index data presented in this study are the averaged Tur indices representing all Tur indices during one cardiac cycle for each observation rather than individual instantaneous values, and may not be the optimal data for the evaluation of post-stenotic flow turbulence. Tur index is a completely direction-based quantification number for turbulent flow. The magnitude information of the velocity has not been considered in quantifying the flow turbulence even though the velocities at different locations within the ROI are different. Current V Flow uses two-dimensional (2D) technology and the Tur index is based on 2D data rather than three-dimensional (3D) data while actual post-stenotic turbulence occurs in a 3D space. A 3D V Flow in the future will deliver a better representation of complex haemodynamic patterns (16).

## Conclusion

This limited study demonstrates the mean Tur index in a cardiac cycle is generally <1% in a straight artery segment without a stenosis. Flow turbulence indicated by increase in the Tur index occurred immediately distal to a stenosis (50 or 70%). The Tur value reaches its peak in the artery around 2.3–3.7 diameters downstream to a stenosis and recovered to <1% earlier in arteries with a less significant stenosis than those with a more significant stenosis. V Flow can provide additional flow turbulence information when assessing arterial stenosis with ultrasound. Further studies with larger sample size are needed to evaluate the value of V Flow in clinical applications.

## Data Availability Statement

The raw data supporting the conclusions of this article will be made available by the authors, without undue reservation.

## Ethics Statement

The animal study was reviewed and approved by the Experimental Animal Welfare and Ethics Committee, the Fourth Medical Center of PLA General Hospital.

## Author Contributions

CW and YT proposed the scientific problems. CW and RZ designed the experiments. RZ, HZ, and WW carried out the animal experiments. RZ and YT processed and calculated the data. YT and RZ conducted the statistical analysis and wrote the draft manuscript. YD provided technical support for the experiment and manuscript writing. All authors contributed to the article and approved the submitted version.

## Funding

This work was supported by the National Natural Science Foundation of China (Grant No. 81771833) and the Beijing Natural Science Foundation (Grant No. 7172209).

## Conflict of Interest

YD is employed by Shenzhen Mindray Bio-Medical Electronics Co., Ltd., Shenzhen, China. The remaining authors declare that the research was conducted in the absence of any commercial or financial relationships that could be construed as a potential conflict of interest.

## Publisher's Note

All claims expressed in this article are solely those of the authors and do not necessarily represent those of their affiliated organizations, or those of the publisher, the editors and the reviewers. Any product that may be evaluated in this article, or claim that may be made by its manufacturer, is not guaranteed or endorsed by the publisher.
