# Corrigendum: Quantitative evaluation of post-stenotic blood flow disturbance in canine femoral artery stenosis model: An early experience with vector flow imaging

**DOI:** 10.3389/fcvm.2022.979992

**Published:** 2022-07-20

**Authors:** Rui Zhao, Haining Zheng, Wei Wang, Yigang Du, Yisha Tong, Chaoyang Wen

**Affiliations:** ^1^Department of Ultrasound, Peking University International Hospital, Beijing, China; ^2^Department of Ultrasound, The Fourth Medical Center of PLA General Hospital, Beijing, China; ^3^Shenzhen Mindray Bio-Medical Electronics Co., Ltd., Shenzhen, China; ^4^Department of Vascular Surgery, Austin Hospital, University of Melbourne, Melbourne, VIC, Australia

**Keywords:** arterial stenosis, Doppler, duplex ultrasound, femoral artery, post-stenotic turbulence, vector flow imaging

In the published article, there was an error in the Section **Materials and Methods**, subsection “*V Flow Ultrasound Assessment of Canine Femoral Artery*.” The equation for the Tur index calculation used by the ultrasound system was incorrectly displayed as:


$$Tur=\left(1-\frac{\sqrt{C^{2}-S^{2}}}{N}\right)\times 100\%$$

The corrected equation appears below:


$$Tur=\left(1-\frac{\sqrt{C^{2}+S^{2}}}{N}\right)\times 100\%$$

The authors apologize for this error and state that this does not change the scientific conclusions of the article in any way. The original article has been updated.

## Publisher's note

All claims expressed in this article are solely those of the authors and do not necessarily represent those of their affiliated organizations, or those of the publisher, the editors and the reviewers. Any product that may be evaluated in this article, or claim that may be made by its manufacturer, is not guaranteed or endorsed by the publisher.

